# Supplementary material for: Gender Differences in Adenine Diet-Induced Kidney Toxicity: The Impact of 17β-Estradiol on Renal Inflammation and Fibrosis
Source: Int J Mol Sci. 2025 Feb 6;26(3):1358. doi: 10.3390/ijms26031358 (PMC11818771; doi:10.3390/ijms26031358)
Supplement: Supplementary file 1 [file ijms-26-01358-s001.zip › ijms-3455880-supplementary.pdf]

Supplementary table S1. Primer sequences for qPCR

Mouse

Gene	*Forward (5'-3')*	*Reverse (3'-5')*
Havcr1	*CTGGAATGGCACTGTGACATCC*	*GCAGATGCCAACATAGAAGCCC*
Ssr1	*GGAGGATGTATCCAGTGAACCAG*	*GCCAACCAGGAACTTCACGATG*
Col1a2	*CAGCTCCAGGAAGACCTCGA*	*GTAACAAGGGTGAGCCTGGC*
Vim	*CAAGCCTGACCTCACTGCTG*	*CACCTGTCTCCGGTACTCGT*
Tgfb	*CCTCACCTCCATGTACCAGAA*	*TGGAAATGACCTTGTCAATGAG*
Tnfa	*CGTCAGCCGATTTGCTATCT*	*CGGACTCCGCAAAGTCTAAG*
Il6	*AGTTGCCTTCTTGGGACTGA*	*TCCACGATTTCCCAGAGAAC*
Ccl2	*CCAGCAAGATGATCCCAATG*	*CTTCTTGGGGTCAGCACAGA*
Emr1	*TCTGGGGAGCTTACGATGGA*	*GAATCCCGCAATGATGGCAC*
Cd68	*GGGGCTCTTGGGAACTACAC*	*GTACCGTCACAACCTCCCTG*
18S rRNA	*GTGGTCTTGGTGTGCTGACC*	*GACAACAAGCTGCGTGAGGA*

Rat

Gene	*Forward (5'-3')*	*Reverse (3'-5')*
Acta2	*TTGTCCACCGCAAATGCTTC*	*AAGGCGCTGATCCACAAAAC*
Col1a2	*CGTCGTGCCTAGCAACATGC*	*AGTTCCCAGTAAGACCAGGG*
Col3a1	*TGGGATGCAACTACCTTGGT*	*AGGTGTAGAAGGCTGTGGAC*
Vim	*CGCCATCAACACCGAGTTCA*	*CTCGGCCAGCAGGATTTTGT*
Ccl2	*GCCAACTCTCACTG AGCCA*	*GCATCTGGCTGAGACAGCAC*
Ccl3	*TGCCCTTGCTGTTCTTCTCT*	*AAAGGCTGCTGGTCTCAAAA*
Cxcl1	*AGACAGTGGCAGGGATTCAC*	*GGGGACACCCTTTAGCATCT*
18S rRNA	*AGTCGGCATCGTTTATGGTC*	*CGCGGTTCTATTTTGTTGGT*
